# The overexpression of actin related protein 2/3 complex subunit 1B(ARPC1B) promotes the ovarian cancer progression *via* activation of the Wnt/β-catenin signaling pathway

**DOI:** 10.3389/fimmu.2023.1182677

**Published:** 2023-05-25

**Authors:** Junning Huang, Haiqin Zhou, Caichun Tan, Shien Mo, Tingji Liu, Yan Kuang

**Affiliations:** Department of Gynecology, First Affiliated Hospital of Guangxi Medical University, Nanning, China

**Keywords:** ovarian cancer, actin related protein 2/3 complex subunit 1B, β-catenin, XAV-939, prognosis, oncogene

## Abstract

**Introduction:**

Ovarian cancer is one of the most fatal malignancies of the female reproductive system. The purpose of this study is to explore the mechanism of Actin Related Protein 2/3 Complex Subunit 1B(ARPC1B) in the progression of ovarian cancer.

**Methods:**

The expressions and prognostic value of ARPC1B in ovarian cancer were identified using the GEPIA database and the Kaplan-Meier Plotter database. The expression of ARPC1B was manipulated to evaluate its impact on the malignant phenotypes of ovarian cancer. The cell proliferation ability was analyzed through CCK-8 assay and clone formation assay. The cell migration and invasion capacity was evaluated through wound healing assay and trans well assay. Mice xenografts were conducted to measure the effects of ARPC1B on tumor development *in vivo*.

**Results:**

Our data suggested that ARPC1B was overexpressed in ovarian cancer, which was correlated with a poorer survival compared to low mRNA expression of ARPC1B in ovarian cancer patients. The overexpression of ARPC1B promoted cell proliferation, migration, and invasion of ovarian cancer cells. Conversely, the knockdown of ARPC1B resulted in the opposite effect. Additionally, ARPC1B expression could activate Wnt/β-catenin signaling pathway. The administration of the β-catenin inhibitor XAV-939 abolished the promotion of cell proliferation, migration, and invasion activities induced by ARPC1B overexpression *in vitro*.

**Conclusions:**

ARPC1B was overexpressed in ovarian cancer and was correlated with poor prognosis. ARPC1B promoted ovarian cancer progression through activation of Wnt/β-catenin Signaling Pathway.

## 1 Introduction

Over the past several decades, significant progress has been made in improving cancer survival rates for most types of cancer (1). However, ovarian cancer remains a significant challenge. As the fifth most common cause of cancer-related death among women and the leading cause of mortality among gynecologic malignancies (1), ovarian cancer presents a critical public health concern. Unfortunately, the absence of specific symptoms and diagnostic biomarkers often leads to late diagnosis, with more than 70% of patients being diagnosed at clinical stage III or IV according to the Federation International of Gynecology and Obstetrics (FIGO) classification system (2). This results in a high mortality rate, with more than 75% of women with advanced ovarian cancer succumbing to the disease. In contrast, when ovarian cancer is diagnosed at an early stage, with the tumor confined to one or two sides of the ovaries, the cure rate can reach 90% (3). The current main therapeutic strategies for ovarian cancer include chemotherapy, surgery, and targeted therapy (4). Despite abundant research on the pathogenesis and therapy for ovarian cancer, there is still a lack of authoritative treatment. Therefore, the identification of effective predictive biomarkers for early diagnosis and personalized treatment is an urgent need in the field of ovarian cancer research.

Actin Related Protein 2/3 Complex Subunit 1B (ARPC1B), also known as ARC41, P41-ARC, P40-ARC, PLTEID, and IMD71, encodes one of seven subunits of the human Arp2/3 protein complex (5). This complex has been implicated in a variety of crucial biological functions, including regulation of cell differentiation, migration, adhesion, and cargo transport (6, 7). Recent studies have demonstrated that ARPC1B promotes cancer cell invasion and metastasis in several types of cancer, including glioblastoma and prostate cancer (8, 9). Additionally, ARPC1B has been linked to radiotherapy resistance, as ARPC1B-deficient patients exhibit increased sensitivity to ionizing radiation and the drug bleomycin (10).Furthermore, the overexpression of ARPC1B has been shown to promote radiotherapy resistance and maintain mesenchymal phenotype in glioma stem cells (11). Despite its known role in other types of cancer, the role of ARPC1B in ovarian cancer has not yet been reported in the literature.

β-catenin, also known as CTNNB1, is the key downstream component of the canonical Wnt/β-catenin signaling pathway (12). This pathway plays a crucial role in tumorigenesis and is activated in many ovarian epithelial carcinomas (13). Upon activation of the Wnt/β-catenin pathway, β-catenin is released from the cell membrane and redistributes to the nuclei and cytoplasm of tumor cells (14). Wnt/β-catenin signaling pathway is associated with tumor proliferation, metastasis, epithelial-to-mesenchymal transition (EMT), recurrence, chemoresistance, and anti-tumor immune regulation (15, 16). As such, the Wnt/β-catenin signaling pathway represent important targets for the development of new therapeutic strategies for ovarian cancer.

In this work, we conducted a bioinformatics analysis and found that the expression of ARPC1B was significantly elevated in ovarian cancer patients. Survival analysis revealed that high expression of ARPC1B was associated with poor overall survival and progression-free survival in these patients. We then explored the effects of modulating ARPC1B expression on ovarian cancer cells and found that it significantly influenced cell proliferation, metastasis, and invasion *in vitro*, as well as the growth of ovarian cancer tumors *in vivo*. Further investigation revealed that these effects were linked to the regulation of the Wnt/β-catenin signaling pathway.

## 2 Materials and methods

### 2.1 Databases and data analysis

The relationship between ARPC1B expression level and overall survival in ovarian cancer was assessed using Gene Expression Profiling Interactive Analysis (GEPIA) and Kaplan-Meier Plotter (KM plotter) databases that include the Gene Expression Omnibus (GEO), European Genome-phenome Archive (EGA), Genotype-Tissue Expression (GTEx), and The Cancer Genome Atlas (TCGA).

### 2.2 Clinical specimens

The study was approved by the Institutional Research Ethics Committee of Guangxi Medical University. In the study, ovarian specimens were collected from patients who were hospitalized in the First Affiliated Hospital of Guangxi Medical University from January 2021 to November 2022. The specimens were taken from both normal and cancer tissues and stored at -80°C for further experiments. All patients signed informed consent forms and none of them received any treatment before surgery. The normal ovarian specimens were taken from patients who underwent adnexectomy for uterine myoma or adenomyosis. The patients were between 18-70 years old with an average age of 46 years and all diagnoses were determined by pathological examination of the ovarian tissues.

### 2.3 Cell culture, cell transfection, and reagents

The ovarian epithelial cell line IOSE80 and ovarian cancer cell lines A2780, CAOV3, and SKOV3 were purchased from the China Center for Type Culture Collection (CCTCC, Wuhan, China). The cells were propagated in RPMI-1640 medium (Procell, Wuhan, China) containing 10% fetal bovine serum (Procell) and incubated at 37°C under a humidified atmosphere containing 5% CO_2_. Plasmid vectors expressing small hairpin RNA (shRNA) targeting ARPC1B were named shRNA1 or shRNA2. The complementary cDNAs of ARPC1B were synthesized and the plasmid overexpressed vectors pLV3-CMV-3×FLAG-CopGFP-Puro (Miaolingbio, Wuhan, China) of ARPC1B were constructed as ov-ARPC1B. The shRNA and scramble control sequences were listed in **Table 1**. The plasmid vectors were transfected into A2780 and SKOV3 cells using Lipofectamine 3000 (Invitrogen, Carlsbad, California, USA) according to the manufacturer’s instructions. To determine the effect of ARPC1B on the Wnt/β-catenin signaling pathway, we used XAV-939 (MedChemExpress, USA), an inhibitor of β-catenin, at a concentration of 5.0 μM.

**Table 1:** The sequences of shRNAs and scramble control.

Group	Sequence
**Scramble control**	5’-CAACAAGATGAAGAGCACCAAT-3’
**shRNA1**	5’-GTGTGATCTCCATCTGTTATT-3’
**shRNA2**	5’-CCAAGGTGCACGAGCTCAAGG-3’

### 2.4 RNA extraction, rt-PCR, and RT-qPCR

The extraction of RNA was performed from ovarian cancer tissue or cell lines using a Total RNA Extraction Kit (Axygen, USA) according to the manufacturer’s protocol. The genomic DNA present in the RNA samples was eliminated, and cDNA was synthesized using an RNA Reverse Transcription Kit (Servicebio, Wuhan, China). Subsequently, RT-qPCR was carried out using 2x Universal Blue SYBR Green qPCR Master Mix (Servicebio) on a CFX Touch Real-Time PCR Machine (Bio-rad, USA). The reaction mixture was subjected to denaturation at 95°C for 30 seconds, followed by 40 cycles of 15 seconds at 95°C, 10 seconds at 55°C, and 30 seconds at 72°C. The expression levels of ARPC1B was quantified using the 2^-ΔΔCt^ method. The primers for ARPC1B and β-actin were synthesized by GeneSys (Nanning, China), and their sequences are listed in **Table 2**.

**Table 2:** The primers for qRT−PCR analysis.

Gene	Primer	Sequence
**ARPC1B**	Forward	5’- GACAAGAAGATGGCCGTCGC -3’
	Reverse	5’- TGCGAGCTCTGCTTAGGAAC -3’
**β-actin**	Forward	5’- CTCAGGATTTAAAAACTGGAACG -3’
	Reverse	5’- GACAAAAAAGGGGGAAGGG -3’

### 2.5 Protein extraction and western blot

Total protein was extracted from ovarian cancer tissue or cell lines as per the manufacturer’s instruction using RIPA (Solarbio, Beijing, China). The protein concentration was quantified using BCA Protein Assay Kit (Beyotime, Shanghai, China), and 40 μg of protein was subjected to electrophoresis on a 10% SDS-PAGE gel. The proteins were then transferred onto a 0.22 μm PVDF membrane (Merck, USA). The protein bands were incubated with primary antibodies at 4°C overnight, after being blocked with Non-Protein Blocking Solution (Servicebio). Subsequently, the membranes were incubated with dylight-800 labeled secondary antibodies (Invitrogen; 1:10000) at 37°C for one hour. The protein probes were visualized on Odyssey CLx (LI-COR, USA). The primary antibodies used were ARPC1B (Proteintech, Wuhan, China; 1:3000), β-tubulin (Proteintech; 1:2000), β-catenin (Servicebio; 1:1000), cyclin D1 (Huabio, Hangzhou, China; 1:2000), c-myc (ABmart, Shanghai, China; 1:500).

### 2.6 Cell counting Kit-8 assay (CCK-8 assay)

In accordance with the manufacturer’s protocol, the Cell Counting Kit-8 (CCK-8) assay was employed to determine the effect of ARPC1B on cell proliferation. Infected cells were plated into 96-well plates and were cultured for 0 hours, 24 hours, 48 hours, 72 hours, and 96 hours. Then, a 10 μL aliquot of the CCK-8 kit (Servicebio) was added to each well and incubated at 37°C with 5% CO_2_ for 2 hours. The optical density (OD) value was measured at 450 nm using a modular multimode microplate reader machine, the Synergy H1 (BioTek, USA).

### 2.7 Colony formation assay

After being infected for 24 h, 500 cells of SKOV3 or 1000 cells of A2780 were seeded in each well of a six-well plate to assess the impact of ARPC1B on cell clonogenesis. The cells were allowed to grow for 10-14 days, forming colonies which were then fixed with methanol and stained with 0.5% crystal violet. The number of colonies was subsequently counted.

### 2.8 Wound healing assay

The cellular migration ability was evaluated using the wound healing assay. After being infected for 24 hours, infected cells were plated into 6-well plates. After 24 hours of cell culture, a wound was achieved in each well by 1 mL pipette tips. The cells were rinsed with a serum-free medium. Photographic documentation of the distance between cells was taken at 0 hours, 24 hours, and 48 hours.

### 2.9 Transwell assay

After being infected for 24 h, infected cells were harvested and placed in the upper chambers of transwell inserts (Corning, USA), with a non-serum medium. In the lower chambers, 900 mL of medium containing 10% FBS was added, and the system was maintained for 48 hours. The cells were then fixed with methanol and stained with 0.5% crystal violet (Servicebio). The number of migrated cells in the lower chambers was quantified to reveal the cell invasion ability.

### 2.10 Tumor xenograft model

Six-week-old BALB/c nude mice (Guangxi Medical University, Nanning, China) were handled and managed in accordance with the agreement approved by Guangxi Medical University. Exponentially growing infected cells (either SKOV3 or A2780 cells) were subcutaneously inoculated into the armpit region of the mice. One week after the injection, the tumor diameter was measured every 4 days and used to calculate the tumor volume. The mice were sacrificed on the 27th day after inoculation and the tumors were then utilized for Western Blot analysis.

### 2.11 Statistical analysis

The statistical analyses in this study were executed utilizing the R software version 4.2.2. The data were represented as the mean and standard deviation, derived from a minimum of three independent experiments. To assess the differences between two groups, the Student’s t-test was employed, whereas, for comparisons among three or more groups, an ANOVA was conducted. A p-value of less than 0.05 was considered as statistically significant.

## 3 Results

### 3.1 The expression of ARPC1B and its co-relation of the overall survival of ovarian cancer

**Figure 1:**
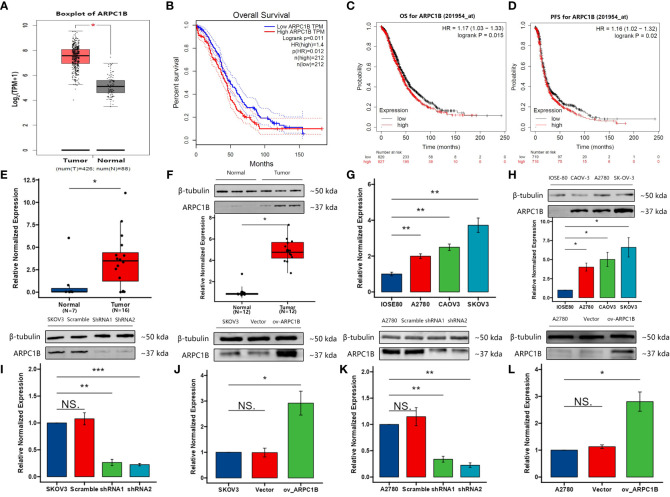
The expression of ARPC1B and its co-relation of the overall survival of ovarian cancer. **(A)** Boxplot of ARPC1B in ovarian cancer. TPM, transcripts per million. **(B)** Overall survival time between patients with high and low ARPC1B expression by GEPIA with median cut-off. Dotted lines indicated the 95% confidence interval. HR, hazard ratio. **(C, D)** Overall survival time and progression-free survival time between patients with high and low ARPC1B expression by KM Plotter with median cut-off. **(E, F)** Relative mRNA and protein expression of ARPC1B in normal ovarian tissues and ovarian cancer tissues. **(G, H)** Relative mRNA and protein expression of ARPC1B in ovarian epithelial cell line IOSE80 and ovarian cancer cell lines A2780, CAOV3, and SKOV3. **(I, J)** Relative protein expression of ARPC1B in infected SKOV3 cells. **(K, L)** Relative protein expression of ARPC1B in infected A2780 cells. Measurement data were expressed as mean ± SD of three independent experiments. NS, Not statistically significant. *p < 0.05, **p < 0.01, ***p < 0.001.

The relationship between ARPC1B expression level and overall survival in ovarian cancer was assessed using Gene Expression Profiling Interactive Analysis (GEPIA) and Kaplan-Meier Plotter (KM plotter) databases that include the Gene Expression Omnibus (GEO), European Genome-phenome Archive (EGA), and The Cancer Genome Atlas (TCGA). Our results showed that ARPC1B was significantly overexpressed in ovarian cancer tissues compared to normal tissues (**Figures 1A**). The survival analysis indicated that a high expression level of ARPC1B was associated with poorer overall survival and progression-free survival in ovarian cancer patients (**Figures 1B–D**), suggesting the prognostic significance of ARPC1B. We determined the mRNA and protein expression of ARPC1B in ovarian tissue and ovarian cells, including ovarian epithelial cell line IOSE80 and ovarian cancer cell lines A2780, CAOV3, and SKOV3. The results demonstrated that ARPC1B was significantly overexpressed in ovarian cancer tissues compared to normal ovarian tissues, and significantly overexpressed in ovarian cancer cell lines compared to ovarian epithelial cell line (**Figures 1E–H**). ARPC1B expression was low in A2780 cells and high in SKOV3 cells (**Figures 1G, H**). We then artificially regulated the expression of ARPC1B in A2780 and SKOV3 cells and confirmed through Western Blot analysis (**Figures 1I–L**).

### 3.2 Effects of ARPC1B on the progression of ovarian cancer cells

**Figure 2:**
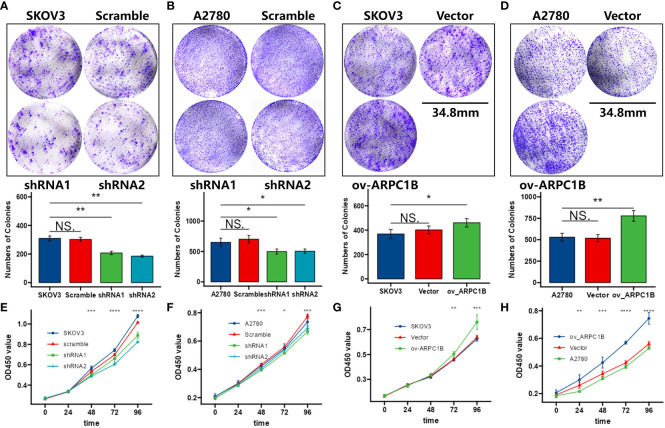
Effects of ARPC1B on the proliferation of ovarian cancer cells. **(A–D)** Cellular clone forming ability was evaluated by colony formation assay. **(E–H)** Cell proliferation ability was assessed by CCK-8 assay. Measurement data were expressed as mean ± SD of three independent experiments. NS, Not statistically significant. *p < 0.05, **p < 0.01, ***p < 0.001.

**Figure 3:**
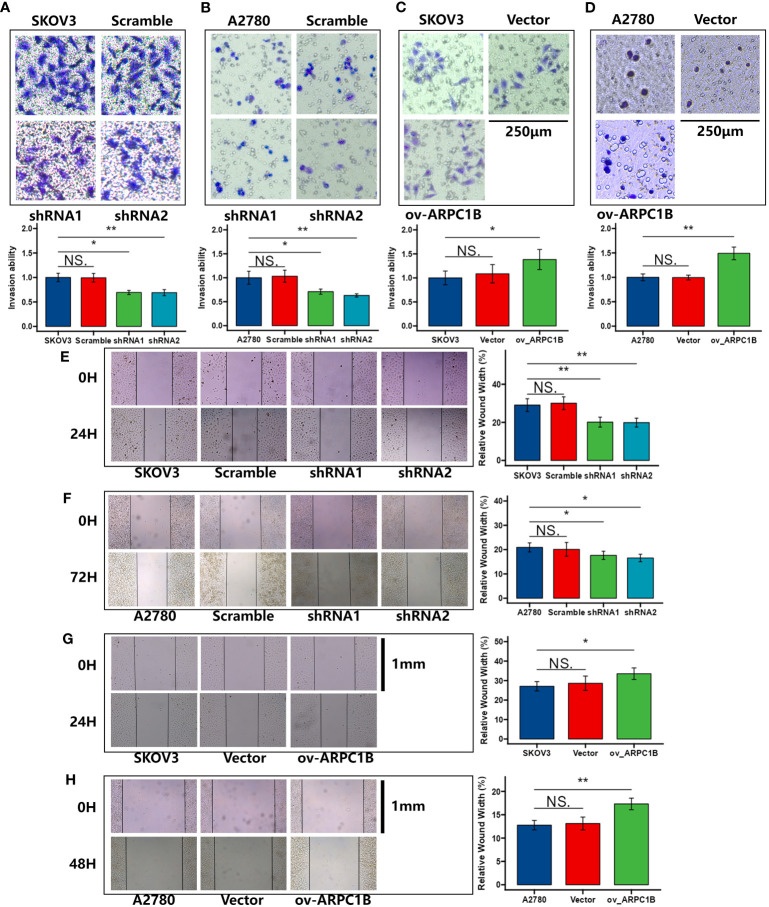
Effects of ARPC1B on the migration of ovarian cancer cells. **(A–D)** Cell invasion ability was detected by transwell assay. **(E–H)** Cell migration ability was assessed by wound healing assay. Measurement data were expressed as mean ± SD of three independent experiments. NS, Not statistically significant. *p < 0.05, **p < 0.01, ***p < 0.001.

Using the clone formation assay and CCK-8 assay, we evaluated the proliferation ability of ovarian cancer cells. The results of the clone formation assay showed that knocking down ARPC1B significantly decreased the proliferation of A2780 and SKOV3 cells, but the effect was weaker in A2780 cells (**Figures 2A, B**). Overexpression of ARPC1B significantly enhanced the proliferation of A2780 and SKOV3 cells, but the effect was weaker in SKOV3 cells (**Figures 2C, D**). The results of the CCK-8 assay were consistent with those of the clone formation assay. Knocking down ARPC1B significantly reduced the proliferation of A2780 and SKOV3 cells, but the effect was weaker in A2780 cells (**Figures 2E, F**). Overexpression of ARPC1B significantly increased the proliferation of A2780 and SKOV3 cells, but the effect was weaker in SKOV3 cells (**Figures 2G, H**). We also assessed the migration ability of ovarian cancer cells using the Transwell assay and wound healing assay. The results of the Transwell assay showed that knocking down ARPC1B significantly reduced the migration ability of A2780 and SKOV3 cells (**Figures 3A, B**), while overexpression significantly increased the migration ability of A2780 and SKOV3 cells, but the effect was weaker in SKOV3 cells (**Figures 3C, D**). The results of the wound healing assay were consistent with those of the Transwell assay. Knocking down ARPC1B significantly reduced the migration ability of A2780 and SKOV3 cells, but the effect was weaker in A2780 cells (**Figures 3E, F**). Overexpression of ARPC1B slightly increased the migration ability of A2780 and SKOV3 cells (**Figures 3G, H**). Overall, our results suggest that knocking down ARPC1B reduces the proliferation and migration ability of A2780 and SKOV3 cells, and this effect is generally more significant in SKOV3 cells than in A2780 cells. On the other hand, overexpression of ARPC1B enhances the proliferation and migration ability of A2780 and SKOV3 cells, and this effect is generally more significant in A2780 cells than in SKOV3 cells.

### 3.3 Effects of ARPC1B on the Wnt/β-catenin signaling pathway in ovarian cancer cells

**Figure 4:**
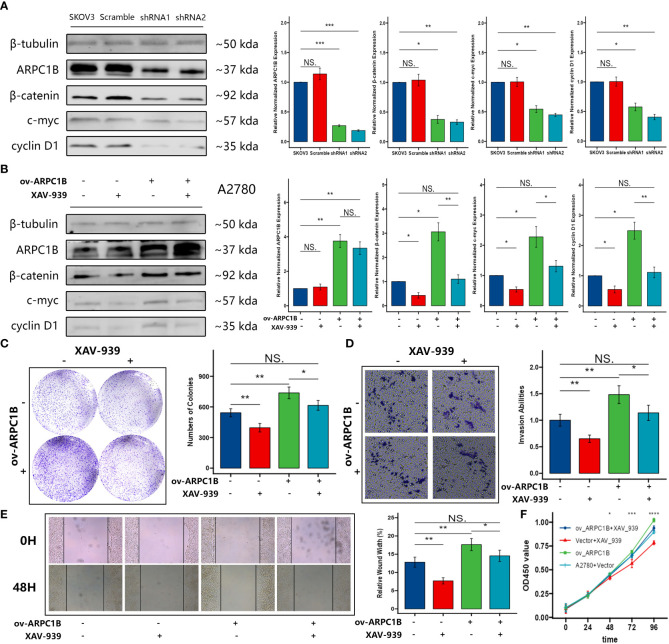
Effects of ARPC1B on the Wnt/β-Catenin signaling pathway in ovarian cancer cells. **(A)** The protein expression of β-catenin, c-myc, cyclin D1 in SKOV3 cells following ARPC1B knockdown. **(B)** The protein expression of β-catenin, c-myc, cyclin D1 in A2780 cells following ARPC1B overexpression. **(C)** Cellular clone forming ability was evaluated by colony formation assay. **(D)** Cell invasion ability was detected by transwell assay. **(E)** Cell migration ability was measured by wound healing assay. **(F)** Cell proliferation ability was assessed by CCK-8 assay. Measurement data were expressed as mean ± SD of three independent experiments. NS, Not statistically significant. *p < 0.05, **p < 0.01, ***p < 0.001.

To further explore the mechanism of the effects caused by ARPC1B, the expression of key proteins (β-catenin, c-myc, and cyclin D1) in the Wnt/β-Catenin signaling pathway was evaluated. Western Blot demonstrated that the knockdown of ARPC1B attenuated the expression of β-catenin in SKOV3 cells, leading to the low expression of c-myc and cyclin D1 (**Figure 4A**). The overexpression of ARPC1B promoted the expression of β-catenin in A2780 cells, causing the up-regulation of c-myc and cyclin D1, and the promotion was abolished by the administration of β-Catenin inhibitor XAV-939 (**Figure 4B**). The results of the colony formation assay, transwell assay, wound healing assay, and CCK-8 showed that ARPC1B overexpression-induced promotion of cellular proliferation and migration was abolished by the administration of β-Catenin inhibitor XAV-939 (**Figures 4C–F**). These data confirmed that the administration of β-Catenin inhibitor XAV-939 could reverse the malignant process caused by the up-regulated ARPC1B in ovarian cancer. Our results suggested the possibility that the overexpression of ARPC1B promoted the expression of cell proliferation or metastasis-related proteins by the activation of the Wnt/β-Catenin signaling pathway in ovarian cancer.

### 3.4 Effects of ARPC1B on the growth of ovarian cancer tumor *in vivo*

**Figure 5:**
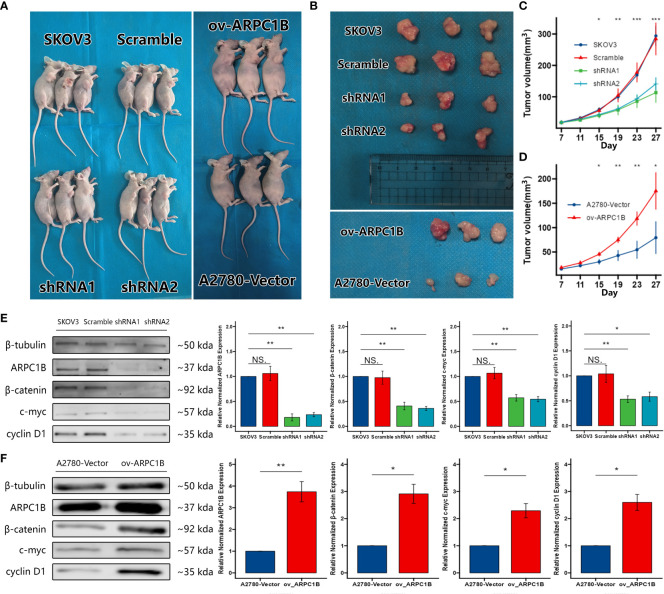
Effects of ARPC1B on the growth of ovarian cancer tumor *in vivo*. Xenograft tumor models were built with infected SKOV3 cells or A2780 cells. **(A–D)** The volume of xenograft tumors. The tumors were extracted on day 27 **(E, F)** Relative protein expression of β-catenin, c-myc, cyclin D1. Measurement data were expressed as mean ± SD of three independent experiments. NS, Not statistically significant. *p < 0.05, **p < 0.01, ***p < 0.001.

To confirm the effects of ARPC1B on the growth of ovarian cancer tumor *in vivo*, xenograft tumor models were built with SKOV3 cells infected with shRNA1 and shRNA2 or A2780 cells infected with ov-ARPC1B. Compared to scramble control, ARPC1B interference markedly ameliorated the average tumor volume. However, compared to vector, the overexpression of ARPC1B significantly increased the average tumor volume (**Figures 5A–D**). Then, we verified the expression of ARPC1B and further proved the association between ARPC1B and Wnt/β-Catenin signaling pathway using Western Blot. The protein expression of β-Catenin was weakened in tumors derived from the infected SKOV3 cells model, causing the attenuated expression of c-myc and cyclin D1 (**Figures 5E**). The protein expression of β-Catenin was elevated in tumors derived from the A2780 cells model, leading to the up-regulation of c-myc and cyclin D1 (**Figures 5F**). The results suggested that the overexpression of ARPC1B promoted the growth of ovarian cancer tumors *in vivo via* the activation of the Wnt/β-Catenin signaling pathway.

## 4 Discussion

Despite a decrease in incidence rates in recent years due to increased use of oral contraceptives (17), ovarian cancer remains the leading cause of death from gynecologic cancer, with a five-year survival rate of only 48% (18). The cure rate of early stage ovarian cancer can reach 90% (3), however, screening for ovarian cancer remains challenging due to its vague and nonspecific symptoms. The positive predictive value of routine screening methods, including ultrasound and serum CA-125, is less than 50% with a false-positive rate of up to 44% (19). Treatment of ovarian cancer typically involves a combination of chemotherapy and surgery, including surgical staging of affected tissue, tumor debulking surgery, subsequent chemotherapy, and target therapy such as PARP inhibitors (20, 21). However, late diagnosis and drug resistance present significant challenges in the treatment of ovarian cancer. To address these challenges, immediate research priorities should focus on developing novel diagnosis marker and therapy for ovarian cancer (22). It is well established that certain genes are associated with an increased risk of ovarian cancer (17, 23). Mutations in the BRCA1, BRCA2, and MMR genes can increase the risk of ovarian cancer from 1.6% to 40%, 18%, and 10%, respectively (24). To identify novel oncogenes in ovarian cancer, we conducted a data mining analysis of public cancer genomics databases.

In this study, we conducted a bioinformatics analysis and found that the expression of ARPC1B was overexpressed in ovarian cancer. Further analysis revealed that patients with high expression of ARPC1B had a poorer overall survival and progression-free survival compared to those with low expression. To investigate the effect of ARPC1B on ovarian cancer progression, we modulated its expression in ovarian cancer cell lines SKOV3 and A2780. The results showed that overexpression of ARPC1B enhanced cell proliferation and migration *in vitro* through activation of the Wnt/β-Catenin signaling pathway. Conversely, knockdown of ARPC1B resulted in the opposite effect. Furthermore, administration of β-Catenin inhibitor XAV-939 was observed to abolish the proliferation and migration induced by ARPC1B overexpression. Our findings *in vivo* confirmed that ARPC1B overexpression facilitated the growth of ovarian cancer xenograft tumors, while ARPC1B interference suppressed tumor growth. This is the first report to suggest that ARPC1B is involved in ovarian cancer progression and may act as an oncogene in ovarian cancer.

The oncogene role of ARPC1B has been observed in other cancers as well. The overexpression of ARPC1B in glioma cells has been shown to maintain the malignant phenotype, including migration, invasion, and epithelial-to-mesenchymal transition (EMT) status (8, 11). On the other hand, knockdown of ARPC1B in prostate cancer cells has been observed to reduce the proliferation, migration, and invasion and cause cell cycle arrest at the G2/M phase *via* the downregulation of AURKA (9). Further research is needed to explore the effects of ARPC1B on the cell cycle in ovarian cancer and its underlying mechanisms. In the field of immunology, the overexpression of ARPC1B has been shown to promote macrophage recruitment through the activation of the NF-κB and STAT3 pathways (8). Neutrophils were defective in actin microfilament reorganization due to a mutation in ARPC1B or inhibition of its upstream regulator, and Rac2 lose their ability to upregulate complement receptor immunoglobulin expression (25). ARPC1B is also associated with radiotherapy resistance, and patients with ARPC1B deficiency have increased sensitivity to ionizing radiation and bleomycin (11). ARPC1B has been found to promote radiotherapy resistance and maintenance of the mesenchymal phenotype in glioma stem cells (12). Additionally, ARPC1B plays a crucial role in metabolism and the upregulation of ARPC1B in the hypothalamic arcuate nucleus has been linked to the improvement of high-fat diet induced hypothalamic inflammation and leptin resistance (26). The exploitation of cancer metabolism is providing new insight into cancer biology and can potentially lead to the development of more effective targeted treatments for patients (27).

ARPC1B encodes one of seven subunits of the human Arp2/3 protein complex (5), which is the only molecular machine that generates branched actin networks (7), and plays a crucial role in the regulation of various biological functions, including cell differentiation, migration, adhesion, as well as cargo transport (6, 7). Immunohistochemical analysis has revealed that Arp2/3 subunits are overexpressed in a number of cancers, including bladder (28), breast (29), colorectal (30), gastric (31), gliomas (32), and lung cancers (33). In this study, we have discovered that ARPC1B promotes the progression of ovarian cancer by activating the Wnt/β-Catenin signaling pathway. However, it is still unclear whether ARPC1B indirectly activates the pathway through the Arp2/3 protein complex or whether it directly activates a known molecule within the pathway. Further research is needed to clarify this mechanism.

Interestingly, our results suggest that knocking down ARPC1B leads to a more significant decrease in the proliferation and migration abilities of ovarian cancer cells in SKOV3 cells than in A2780 cells. Conversely, overexpression of ARPC1B leads to a more significant increase in the proliferation and migration abilities of ovarian cancer cells in A2780 cells than in SKOV3 cells. We hypothesize that this difference may be due to the characteristics of the cells, as A2780 cells are derived from primary tissues of ovarian adenocarcinoma, while SKOV3 cells are derived from ascites fluids of patients with ovarian serous carcinoma (34, 35). A wound healing assay involving 10 types of ovarian cancer cell lines showed that at 30 hours, the healing rate of SKOV3 cells could reach over 80%, while the healing rate of A2780 cells was less than 20% (36). This suggests that active cell lines like SKOV3 may be more suitable as models for observing anticancer effects, while less active cell lines like A2780 may be more suitable as models for observing cancer-promoting effects. Alternatively, adjusting the observation time points flexibly may be necessary to ensure that the most significant differences between different treatment groups can be observed. However, this speculation is based on limited experimental evidence and cannot completely rule out the possibility of accidental circumstances. We also concern the involvement of other proteins in a compensation process, which could mask the effects of ARPC1B. Additionally, the effects observed *in vivo* appear to be clearer than those observed *in vitro*, suggesting that cellular interactions between tumor cells and the tumor microenvironment (TME) may play a role *in vivo* that is not observable *in vitro*. We will continue to pay attention to these issues in future research.

In summary, this study advances our knowledge of the molecular pathogenesis of ovarian cancer. Further research is needed to fully understand the mechanism by which ARPC1B activates the Wnt/β-catenin signaling pathway in ovarian cancer cells and to assess the potential therapeutic value of targeting ARPC1B. Future studies should focus on examining a greater number of tissue samples using immunohistochemistry to further validate the diagnostic value of ARPC1B and exploring the impact of ARPC1B on the tumor microenvironment, metabolism, drug resistance, and radiotherapy resistance in ovarian cancer.

## 5 Conclusion

The expression levels of ARPC1B were found to be elevated in ovarian cancer tissues. The overexpression of ARPC1B has been shown to contribute to the malignant phenotype in ovarian cancer *via* activation of the Wnt/β-Catenin signaling pathway. These findings suggest that ARPC1B may be a novel target in the arsenal to treat ovarian cancer.

## Data availability statement

The raw data supporting the conclusions of this article will be made available by the authors, without undue reservation.

## Ethics statement

The studies involving human participants were reviewed and approved by Institutional Research Ethics Committee of Guangxi Medical University. The patients/participants provided their written informed consent to participate in this study. The animal study was reviewed and approved by Institutional Research Ethics Committee of Guangxi Medical University.

## Author contributions

JH conceived and designed the experiments. JH, HZ, CT, and SM performed the experiments. JH, HZ, CT, SM, TL, and YK collected the clinical specimens. JH performed the statistical analysis. JH wrote the manuscript. YK made revision of the manuscript. All authors contributed to the article and approved the submitted version.
