# Supplementary figures and images for: The overexpression of actin related protein 2/3 complex subunit 1B(ARPC1B) promotes the ovarian cancer progression via activation of the Wnt/β-catenin signaling pathway
Source: Front Immunol. 2023 May 25;14:1182677. doi: 10.3389/fimmu.2023.1182677 (PMC10247967; doi:10.3389/fimmu.2023.1182677)

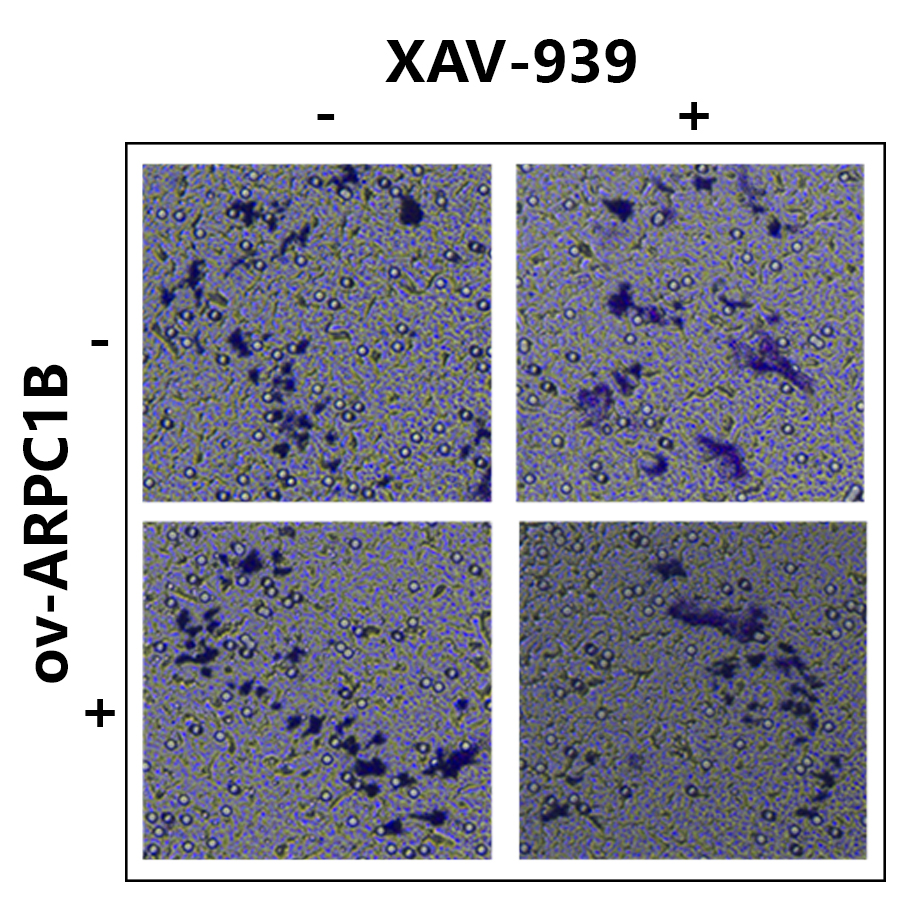

Supplement: Supplementary file 1 [file Image_1.jpeg]

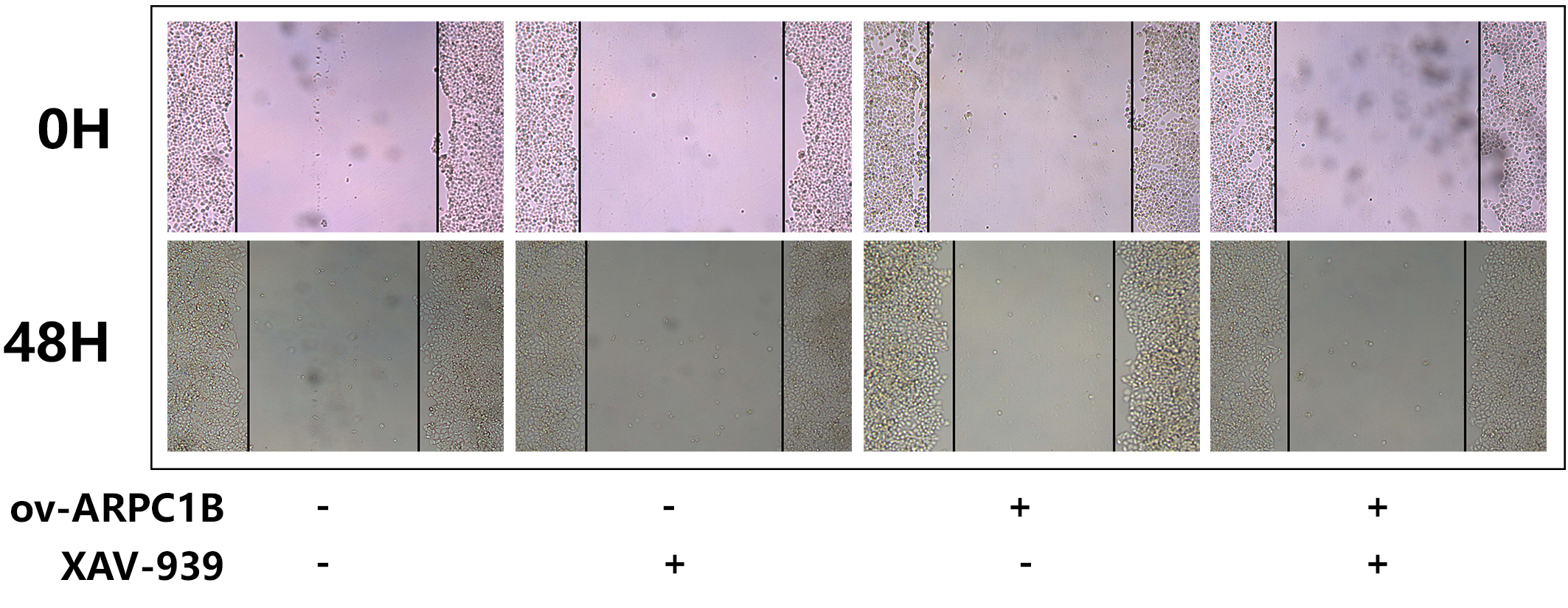

Supplement: Supplementary file 2 [file Image_2.jpeg]

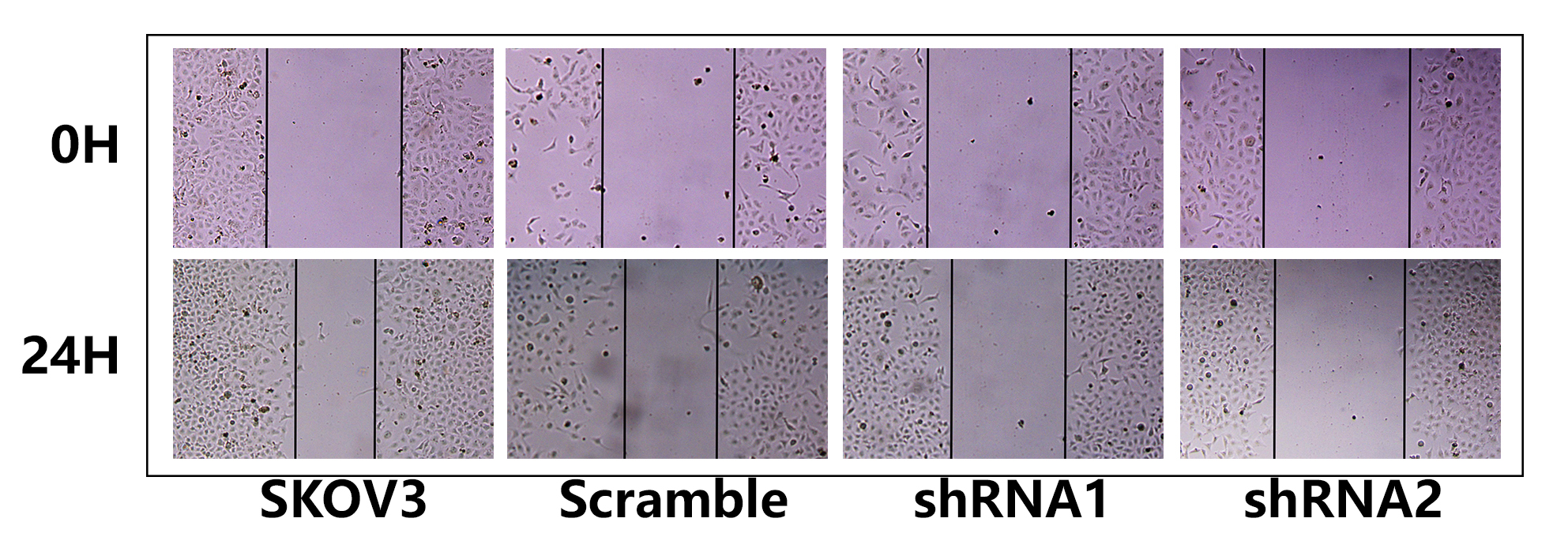

Supplement: Supplementary file 3 [file Image_3.jpeg]

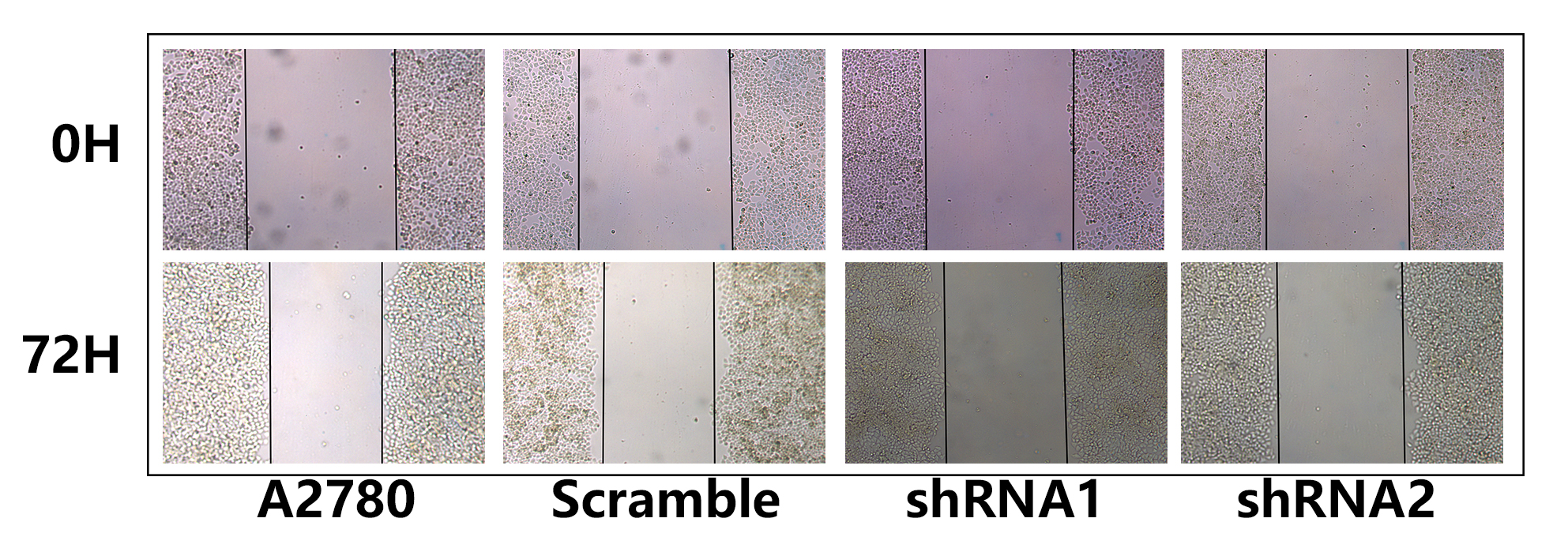

Supplement: Supplementary file 4 [file Image_4.jpeg]

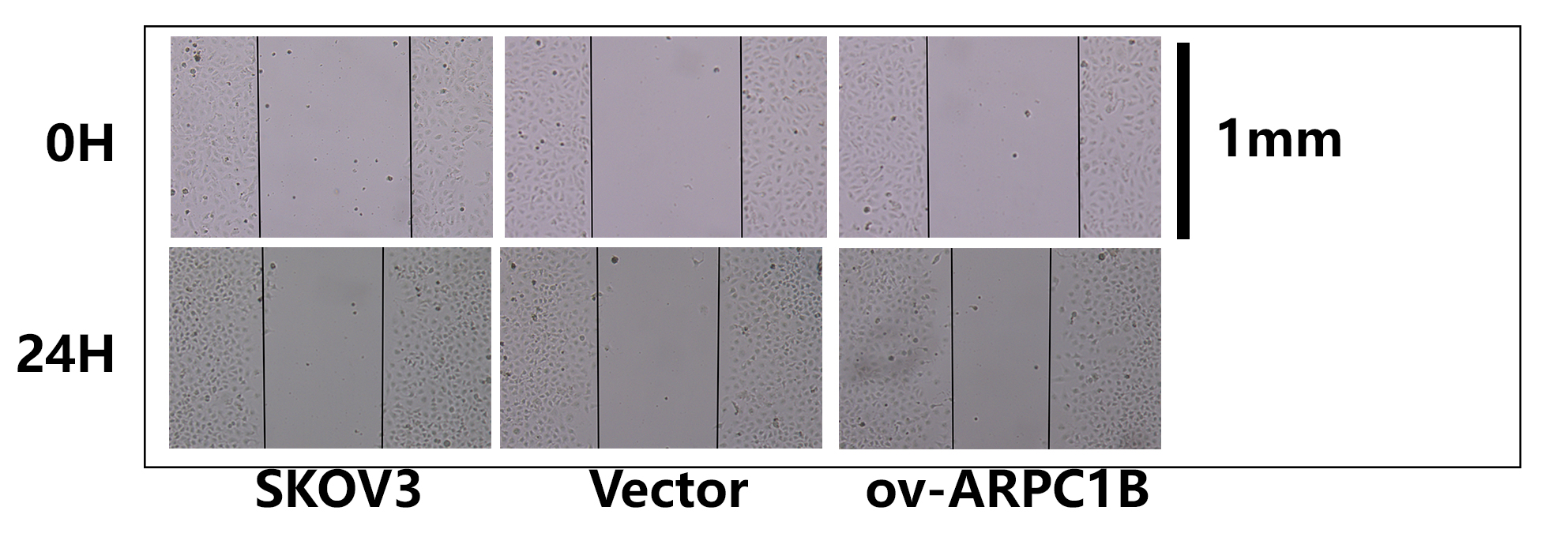

Supplement: Supplementary file 5 [file Image_5.jpeg]

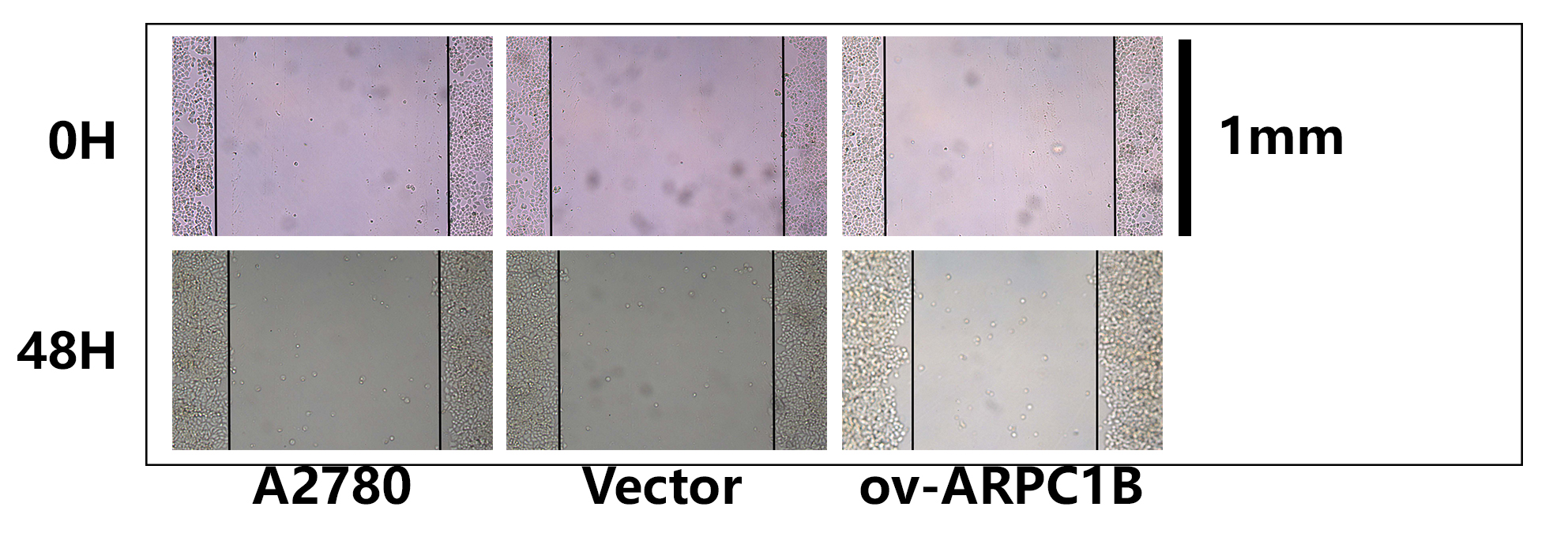

Supplement: Supplementary file 6 [file Image_6.jpeg]
